# Precocious myelination in a mouse model of autism

**DOI:** 10.1038/s41398-019-0590-7

**Published:** 2019-10-07

**Authors:** Maryam Khanbabaei, Elizabeth Hughes, Jacob Ellegood, Lily R. Qiu, Raven Yip, Jenna Dobry, Kartikeya Murari, Jason P. Lerch, Jong M. Rho, Ning Cheng

**Affiliations:** 1 Developmental Neurosciences Research Program, Alberta Children’s Hospital Research Institute (ACHRI), Cumming School of Medicine, University of Calgary, Calgary, AB Canada; 2 Mouse Imaging Centre (MICe), Hospital for Sick Children, Toronto, ON Canada; 3 Department of Electrical and Computer Engineering, Schulich School of Engineering, University of Calgary, Calgary, AB Canada; 4 Departments of Pediatrics, Clinical Neurosciences, Physiology & Pharmacology, Alberta Children’s Hospital Research Institute and Hotchkiss Brain Institute, Cumming School of Medicine, University of Calgary, Calgary, AB Canada; 5 Present Address: University of California, San Diego; Division of Pediatric Neurology, Rady Children’s Hospital San Diego, California, USA

**Keywords:** Neuroscience, Physiology

## Abstract

Autism spectrum disorder (ASD) has been hypothesized to be a result of altered connectivity in the brain. Recent imaging studies suggest accelerated maturation of the white matter in young children with ASD, with underlying mechanisms unknown. Myelin is an integral part of the white matter and critical for connectivity; however, its role in ASD remains largely unclear. Here, we investigated myelin development in a model of idiopathic ASD, the BTBR mice. Magnetic resonance imaging revealed that fiber tracts in the frontal brain of the BTBR mice had increased volume at postnatal day 6, but the difference reduced over time, reminiscent of the findings in young patients. We further identified that myelination in the frontal brain of both male and female neonatal BTBR mice was increased, associated with elevated levels of myelin basic protein. However, myelin pattern was unaltered in adult BTBR mice, revealing accelerated developmental trajectory of myelination. Consistently, we found that signaling of platelet-derived growth factor receptor alpha (PDGFRα) was reduced in the frontal brain of neonatal BTBR mice. However, levels of microRNA species known to regulate PDGFRα signaling and myelination were unaltered. Together, these results suggest that precocious myelination could potentially contribute to increased volume and connectivity of the white matter observed in young children with ASD.

## Introduction

Autism spectrum disorder (ASD) affects multiple brain regions and probably involves impaired connectivity among them^1–4^. Consistent with altered connectivity, abnormal white matter growth and reduced white matter integrity have been reported in patients with ASD^1,3,5^, including atypical development of white matter microstructure observed in young patients^6–15^. In addition, white matter maturation is associated with development of social cognition in early childhood^16^, and altered white matter connectivity probably contributes to core symptoms of ASD including atypical social communication^17^.

Although changes during early development may be critical to the pathogenesis of ASD, they may disappear, or may even be reversed later in life^18^. A notable example is that children with ASD have increased brain size at early childhood, but in older children and adults, decreases in structural volumes are observed instead^19–25^. In a related vein, reduced size of the corpus callosum in children and adults with ASD has been a consistent finding^26^; however, a recent study of infants <2 years of age showed that the corpus callosum was larger in those who later developed ASD^27^. Together, these observations strongly suggest that alterations are dynamic but not uniform across the lifespan of ASD patients, and developmental trajectory could be a critical component of the etiology.

In terms of functional and structural connectivity, evidence suggests that hyper-connectivity may be more characteristic of young children with autism, while hypo-connectivity may be more prevalent in adolescents and adults^18^. In older children, adolescents, and adults with ASD, restriction of water diffusion (often measured by fractional anisotropy, FA, using neuroimaging methods) in white matter tracts is usually reduced, suggesting functional hypo-connectivity. On the other hand, recent imaging studies in patients 1–4-years-old have reported that they display greater volume and increased FA of fiber tracts in mostly frontal regions of the brain, but a slower rate of continued development^6–15^.

The mechanisms underlying altered trajectory of white matter development remain unclear, especially regarding the involvement of the myelin sheath, a fundamental component of the white matter. Myelin formation continues even decades after birth in the human brain. It enables saltatory conduction of action potentials along myelinated axons and regulates the timing of information integration in neural networks^28^. In addition, myelin also provides trophic and metabolic support to the axons^29^. During development, the timing of myelination is critical as myelin stabilizes axon structure and wiring pattern in neural networks. However, due to the limitations of neuroimaging methods and the dearth of studies using animal models (a few such studies so far have shown delayed myelination in general using animal models), development of myelin during the course of ASD has largely remained unknown^30^.

Here, we investigated myelin development in a mouse model of idiopathic ASD, the BTBR *T*^+^ *Itpr3*^*tf*^/J (BTBR) inbred strain. In addition to robust behavioral phenotype that models core autism-like impairments^31–35^, a striking anatomical feature of the BTBR mice is agenesis of the corpus callosum^36–38^. Dysgenesis of the corpus callosum has been considered a major risk factor for ASD^39^, and atypical developmental trajectory of the size and microstructure of the corpus callosum has been one of the most replicated neuroimaging findings in the patient population^26,27^. Here, magnetic resonance imaging (MRI) revealed that fiber tracts in the frontal brain of the BTBR mice had increased volume at postnatal day 6, but the difference reduced over time, reminiscent of the findings in young patients. We further identified accelerated myelination during the neonatal stage, compared with other inbred lines. We observed this phenomenon in both male and female BTBR mice, and found that it involves increased levels of myelin basic protein (MBP) and reduced platelet-derived growth factor receptor alpha (PDGFRα) signaling.

## Methods and materials

### Animals

Breeder C57BL/6 J (B6) and BTBR animals were obtained from the Jackson Laboratory (ME) and the lines were maintained at the mouse facility of the Cumming School of Medicine, University of Calgary. Mice were housed in a humidity- and temperature-controlled room with a 12-h light/dark cycle and were fed ad libitum. Both neonatal (between postnatal day 2 to day 12, specified in the results section for individual experiment), adolescent (P35), and adult (8 to 10 week old) B6 and BTBR male animals were used. Female neonatal B6 and BTBR mice were also examined. The 129S6 (129) inbred strain was originally purchased from Taconic (NY). Experiments on BTBR mice and B6 or 129 mice were performed in parallel. All procedures in this study were performed in accordance with the recommendations from the Canadian Council for Animal Care. The protocol for this study was approved by the Health Sciences Animal Care Committee of the University of Calgary.

### MRI analysis

#### Preparation of brains for scanning

Initially the mice are anesthetized with ketamine/xylazine and intracardially perfused with 30 ml of 0.1 M PBS containing 10 U/ml heparin (Sigma) and 2 mM ProHance (a Gadolinium contrast agent) followed by 30 ml of 4% paraformaldehyde (PFA) containing 2 mM ProHance. Perfusions were performed with a minipump at a rate of approximately 1 ml/min. After perfusion, mice were decapitated and the skin, lower jaw, ears, and the cartilaginous nose tip were removed. The brain and remaining skull structures were incubated in 4% PFA + 2 mM ProHance overnight at 4 °C then transferred to 0.1 M PBS containing 2 mM ProHance and 0.02% sodium azide for at least 7 days prior to MRI scanning.

#### Image acquisition

Images were acquired on a 7 Tesla MRI scanner (Varian Inc., Palo Alto, CA)^40,41^. The contrast required for image registration and assessment of volume is not sufficient with our typical T2-weighted imaging sequence. Therefore, diffusion-weighted imaging was performed to enhance the contrast between white and gray matter to aid in the registration and volume measurements.

#### Diffusion imaging sequence

The diffusion sequence uses an in-house custom built 16-coil solenoid array to acquire images from 12 brains in parallel^42^. The diffusion sequence used was a 3D diffusion-weighted FSE, with TR = 270 ms, echo train length = 6, first TE = 30 ms, TE = 10 ms for the remaining 5 echoes, one average, FOV = 25 mm × 14 mm × 14 mm, and a matrix size of 450 × 250 × 250, which yielded an image with 56 µm isotropic voxels. One *b* = 0 s/mm^2^ image was acquired and 6 high *b*-value (*b* = 2147 s/mm^2^) images were acquired in the following directions (1, 1, 0), (1, 0, 1), (0, 1, 1), (−1, 1, 0), (−1, 0, 1), and (0, 1, −1) corresponding to (G_x_, G_y_, G_z_). Total imaging time was ~14 h.

#### Registration and analysis

To visualize and compare the mouse brains for anatomical volume assessment, the 6 high *b*-value images were averaged together to make a high contrast image necessary for accurate registration. Then these images were linearly (6 parameter followed by a 12 parameter) and nonlinearly registered together. All scans were then resampled with an appropriate transform and averaged to create a population atlas representing the average anatomy of the study sample. All registrations were performed using a combination of the mni_autoreg tools^43^ and ANTS^44^. The result of the registration was to have all scans deformed into exact alignment with each other in an unbiased fashion. For the volume measurements, this allowed for the analysis of the deformations needed to take each individual mouse’s anatomy into this final atlas space, the goal being to model how the deformation fields relate to genotype^40,45^. The Jacobian determinants of the deformation fields are then calculated as measures of volume at each voxel. These measurements were examined on a voxel-wise basis in order to localize the differences found within regions or across the brain. Multiple comparisons were controlled for by using the False Discovery Rate (FDR)^46^.

### Fluorescent immunohistochemical staining and image acquisition

Brain tissue was processed and a free-floating staining method was used according to previously described methods^47^. Briefly, after cardiac perfusion, brains were removed and post-fixed, embedded in gelatin, cryoprotected in 30% sucrose, sectioned in the coronal or sagittal plane using a cryostat. Sections were then incubated with primary (anti-MBP, Millipore, MAB386, 1:500; anti-CNPase, Developmental Studies Hybridoma Bank, 531796, 1:500; anti-PDGFRα, BD biosciences, Clone APA5, 558774, 1:500; anti-Olig2, R&D Systems, AF2418, 1:50; anti-CC1, Millipore, OP80, 1: 2,000) and corresponding secondary antibodies^47^, as well as DAPI, before being mounted onto slides. Images were then acquired with a Zeiss LSM 510 confocal microscope or a Leica SP8 confocal microscope. For an overview of myelination, tile scans using a ×20 lens were conducted and the images were stitched together using Zen software (Zeiss).

### Cell counting

Z-stack confocal images spanning 20 μm depth were collapsed and PDGFRα-positive cells were counted using Imaris from both P2 and P8 animals in the following three brain areas: dorsal lateral striatum, somatosensory cortex, and external capsule, as the BTBR mice do not have crossing corpus callosum. Four to six collapsed image stacks from each brain region of each animal were used. CC1-positive cells were imaged and counted in external capsule region from P8 mice. Four to six images from each animal were used.

### Western blot

Brain regions of interest from mice of indicated ages were dissected on ice and homogenized in RIPA buffer (Pierce Biotechnology, MA) with protease and phosphatase inhibitor cocktails (Roche, QC). Homogenate was centrifuged at 9391 × *g* for 10 min at 4 °C. In all, 10–20 μg of the protein extract was separated by SDS–PAGE, and transferred to polyvinylidene fluoride membranes. After blocking, blots were incubated with primary and corresponding secondary antibodies, and visualized with an enhanced chemiluminescence detection system. Bands were imaged and quantified using a ChemiDOC MP gel imaging system (Bio-Rad, CA). The following primary antibodies were used: PDGFA, Santa Cruz, 1:100; PDGFRα, Santa Cruz, sc-338, 1:200; MBP, Millipore, MAB386, 1:100 (neonatal tissue) or 1:500 (adult tissue); PLP, Santa Cruz, sc-98781, 1:400; actin, Cell Signaling, 4967, 1:10,000. The relative expression levels of a protein were quantified by normalization using actin levels. When quantifying western blot results, full size images without any saturation were used. In addition, images of longer exposure time were taken to ensure that the location of even the weakest band was clear when drawing the region of interest for analysis.

### Quantitative RT-PCR

RNA was isolated from striatum using RNeasy mini kit (Qiagen). cDNA was synthesized from 1 μg of total RNA using an iScript gDNA Clear cDNA Synthesis Kit (Bio-Rad). qRT-PCR was performed using 10 ng of cDNA in a 20-μL reaction using SsoFast EvaGreen Supermix (Bio-Rad). Primer pairs (IDT) were used at a concentration of 0.375 μM. qPCR was performed in duplicate using the following protocol: 95 °C 2 min, 40 cycles of 95 °C 15 s and 60 °C 30 s, and then 75 °C 10 s, followed by a melt curve procedure on a Bio-Rad CFX96 qPCR machine. Cycle thresholds (Ct) were determined by the software CFX manager (Bio-Rad). Primer sequences used for qRT-PCR to quantify mRNA levels of major myelin-related genes: CNP For_ TTTACCCGCAAAAGCCACACA; CNP Rev_ CACCGTGTCCTCATCTTGAAG; MBP For_ GACCATCCAAGAAGACCCCAC; MBP Rev_GCCATAATGGGTAGTTCTCGTGT; PLP 1 For_ CCAGAATGTATGGTGTTCTCCC; PLP 1 Rev_ GGCCCATGAGTTTAAGGACG. Primer sequences of house keeping genes: HRPT1 For_ GCTGACCTGCTGGATTACAT; HRPT1 Rev_ TTGGGGCTGTACTGCTTAAC; Ppia For_ AGCTCTGAGCACTGGAGAGA; Ppia Rev_ GCCAGGACCTGTATGCTTTA; Rpl13a For_ ATGACAAGAAAAAGCGGATG; Rpl13a Rev_ CTTTTCTGCCTGTTTCCGTA.

For microRNA quantification, total RNA was isolated from brain samples using miRCURY RNA isolation kit (Exiqon) according to the manufactures instructions using the modified protocol for fatty tissue. cDNA was synthesized from 10 ng total RNA using a universal cDNA synthesis kit (Exiqon). qRT-PCR was performed using 0.05 ng of cDNA in a 10-μL reaction using Exilent syber green (Exiqon). LNA primer mixes (Exiqon) were used. qPCR was performed in duplicate using the following protocol: 95 °C 10 min, 40 cycles of 95 °C 10 s and 60 °C 60 s, and then 60 °C 5 s, followed by a melt curve procedure on a Bio-Rad CFX96 qPCR machine.

Geometric mean of the Ct values of the housekeeping genes were calculated using the program BestKeeper^48^, to obtain reference Ct values. The relative abundance of transcript of genes of interest was then analyzed using the REST software^49^.

### Transmission electron microscopy, image collection, and computation of the g-ratio

Brain areas of interest were rapidly dissected. A portion of the optic nerve anterior to the optic chiasm was used. Dissected tissue was then immersed in 2% paraformaldehyde and 2.5% glutaraldehyde in 0.1 M cacodylate buffer at pH 7.4 for 2 h at 4 °C. After washing three times with the same buffer, the samples were post-fixed in 1% osmium tetroxide buffered with cacodylate for 1 h at room temperature, dehydrated through a graded series of ethanol and embedded in Spurr’s resin. Thick sections were cut, stained with 1% toluidine blue and examined using a light microscope in order to select appropriate areas. Ultrathin sections were cut with a Leica EM UC7 ultramicrotome using a diamond knife and stained with 2% aqueous uranyl acetate and Reynolds’s lead citrate. Optic nerve, external capsule (BTBR mice do not have crossing corpus callosum) and dorsal striatum at indicated ages were analyzed. Samples were observed using a Hitachi H7650 transmission electron microscope at 80 kV. Images were taken through an AMT600 digital camera mounted on the microscope. Images were collected from multiple (more than 3 for optic nerve at P9 and more than 7 for corpus callosum and striatum at adulthood), randomly chosen locations of each sample.

To quantify the relative thickness of the myelin sheath, g-ratios were computed for individual axons^50^. Both the inner perimeter around the axon and the outer perimeter around the intact myelin sheath were outlined manually using ImageJ and a custom code was used to quantify the pixels in the inner and outer perimeters. Then the inner axonal diameter and the total outer diameter including the myelin sheath were computed and the ratio of the two (g-ratio) was plotted against axonal diameter. This procedure allowed for inclusion of irregularly shaped axons and helped to eliminate bias based on circularity^50^. Totally, 316 axons in corpus callosum and 280 axons in striatum of four B6 mice, and 346 axons in corpus callosum and 291 axons in striatum of four BTBR mice were analyzed. Experimenters who counted myelinated axons or outlined axonal and myelin sheath perimeter were blind to the genotype of the samples.

### Statistical analysis

All sample sizes (*n*) are numbers of animals used. Sample sizes were determined based on similar published studies and the observed results. Student’s *t*-test was performed to determine statistical significance between the sets of BTBR and B6 data, assuming two-tailed distribution and two-sample unequal variance. Distribution normality was calculated using Shapiro–Wilk test. If the data were not normally distributed, Mann–Whitney *U* test was used instead. Values represented the mean ± SEM, except the qRT-PCR results, which were represented by box and whisker plots generated by the REST program. Two-way repeated measure ANOVA followed by post hoc Tukey’s test was used to compare distribution of axonal area in the optic nerve between the B6 and BTBR mice. One-way ANOVA or one-way ANOVA on ranks (when normality test failed) followed by post hoc test was used to compare relative expression levels of MBP and PLP among BTBR, B6, and 129 strains of mice. *P < 0.05; **P < 0.01; ***P < 0.001.

## Results

### Increased volume of white matter tracts in frontal brain regions of the BTBR mice during neonatal development

**Fig. 1:**
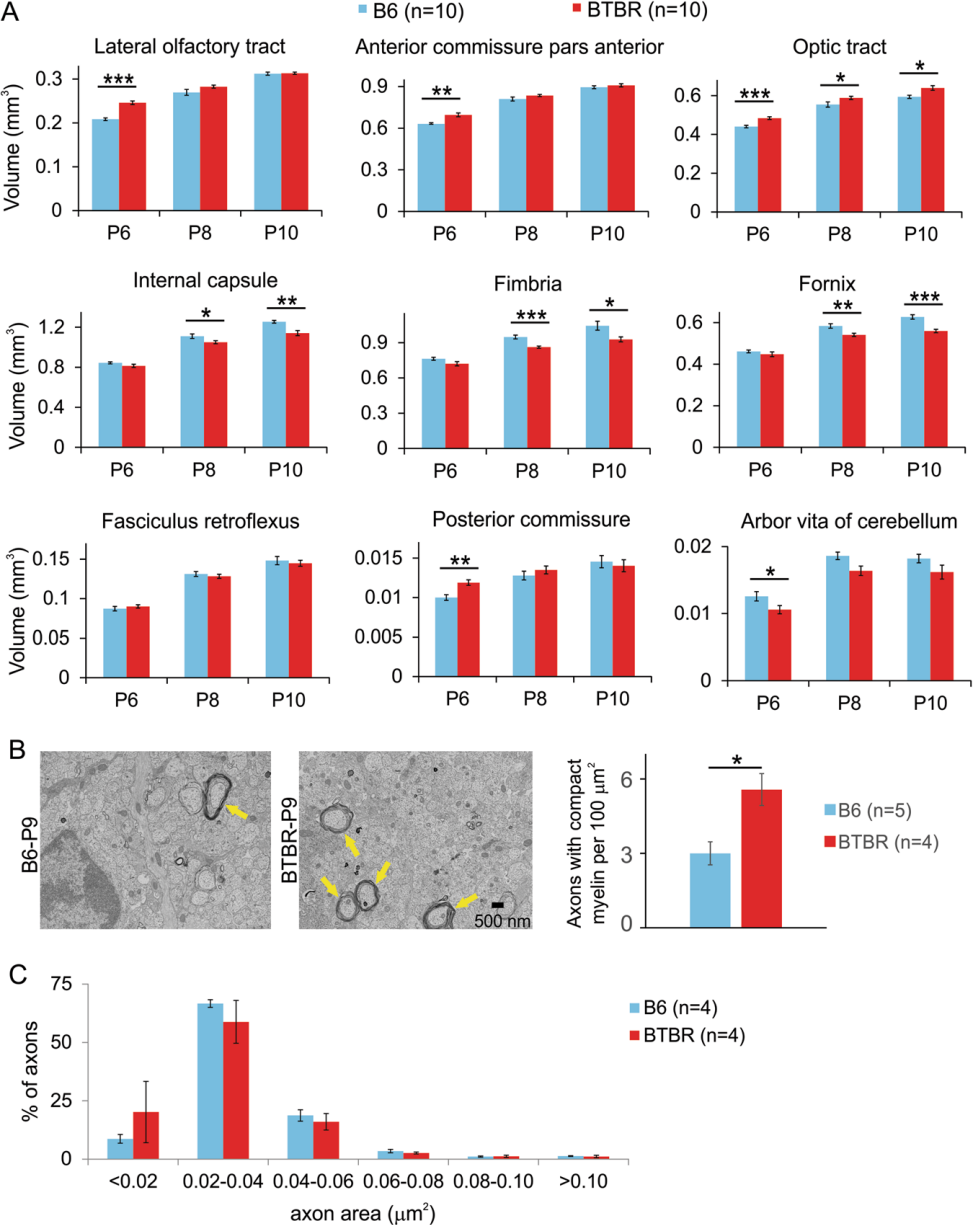
Increased volume of white matter tracts in the frontal brain of the BTBR mice during neonatal development. **a** Comparison of mean volume of white matter tracts at P6, P8, and P10 between B6 and BTBR mice using MRI. Results revealed that fiber tracts in frontal brain regions of the BTBR mice had greater volume at P6, but the difference reduced over time in two out of three cases. *n* = 10 mice for each strain at each age. **b** Representative TEM images of the optic nerve from B6 or BTBR mice at P9, and quantification of axons with compact myelin in optic nerve at this age. Totally, 208 images from five B6 mice and 153 images from four BTBR mice were analyzed. Yellow arrows indicate examples of axons wrapped by compact myelin. **c** Distribution of axonal cross-section area across different size bins in B6 and BTBR optic nerve. Totally, 8951axons from four B6 mice and 10,182 axons from four BTBR mice were analyzed

We first explored whether atypical development of white matter, similar to that in human patients, was also observed in the BTBR model. To this end, we measured volumes of white matter tracts in neonatal male BTBR brains at postnatal day 6 (P6), P8 and P10 using magnetic resonance imaging (MRI). The results were compared with that from age-matched male B6 mice, which display normal levels of sociability and repetitive behaviors and are most often used as the control strain for the BTBR mice in autism-related studies^31–35^. Brains of ten mice of each strain at each age were imaged and analyzed. Results revealed that at P6, four (lateral olfactory tract, anterior commissure pars anterior, optic tract, and posterior commissure) out of 13 white matter tracts had significantly increased volumes in the BTBR mice (false discovery rate all <0.007), while the volumes of the other fiber tracts were not statistically different from those in the B6 mice, with most displaying a trend of decreased volume (Fig. 1a). At P8 and P10, the increase in the volume of lateral olfactory tract, anterior commissure pars anterior, and posterior commissure was reduced so that the BTBR and B6 brains had similar volumes of these tracts. The increase in the volume of the optic tract in the BTBR brain stayed relatively constant for this period. On the other hand, other fiber tracts displayed decreased or similar volumes in the BTBR brain at these ages (Fig. 1a). Note that the corpus callosum in the BTBR mice does not cross the midline^36–38^, but instead forms the Probst bundle, and started to display significantly decreased volume from P8. Together, these data revealed that during this short period of early development, white matter tracts in the BTBR brain exhibited spatially and temporally dynamic alterations compared with the B6 brain: those located more anteriorly displayed an initial increase in volumes, which reduced rapidly in two out of three cases; whereas those located more posteriorly had similar or decreased volumes, except that posterior commissure showed an initial increase at P6 (Fig. 1a). This is reminiscent of the observations from young children with ASD that show increased volume of fiber tracts in mostly frontal regions of the brain, but a slower rate of continued development, resulting in similar volumes of these fiber tracts in older children^6–15^.

### Increased myelination during neonatal development in the frontal brain of both male and female BTBR mice

Next, we examined whether greater volume of anterior fiber tracts in neonatal BTBR brain could be due to increased myelination. Results from MRI indicated that the optic tract in the BTBR mice had a greater volume than that in the B6 mice from P6 to P10 (Fig. 1a). Thus, we first examined the optic nerve at P9 using electron microscopy and quantified axons with compact myelin in cross sections. Results revealed that BTBR mice had significantly greater number of myelinated axons than the B6 mice (Fig. 1b), indicating increased myelination could contribute to the increased volume of the optic tract in the BTBR mice. As axonal size influences initiation of myelination in the optic nerve^51^, we measured cross sectional area of axons in the optic nerve and compared the distribution of axonal area between the B6 and BTBR mice. Results indicated that there was a trend of an increased proportion of the smallest axons and decreased proportions of mid-sized ones in the BTBR mice, but no significant difference between the two strains at any size range we compared (Fig. 1c), suggesting that increased volume of the optic tract in the BTBR mice was not due to greater volume of individual axons, and that size of the axons probably did not contribute to the increased myelination in this model.

**Fig. 2:**
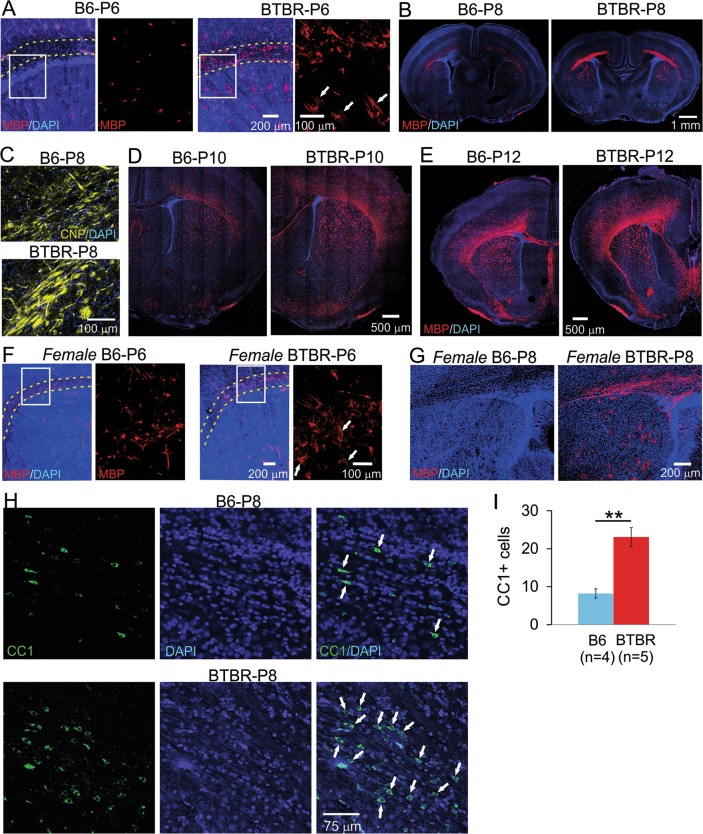
Increased myelination during neonatal development in the frontal brain of both male and female BTBR mice. **a** At P6, MBP signal was located in many cells in male BTBR frontal brain (right panels), including corpus callosum and dorsal striatum, and to a less extent, the cortex. Most of the positive cells had MBP signal in both cell bodies and numerous processes, with some already aligned in parallel tube-like structures presumably wrapping around axons. On the contrary, only a few MBP-positive cells could be identified in the same brain region in the B6 brain (left panels), with the signal mostly restricted to the cell bodies. Dotted lines outline corpus callosum. Boxed region is shown in higher magnification in the right panel next to it. (**b**) At P8, MBP signal in the BTBR frontal brain showed clear organization of myelin sheath in the corpus callosum and dorsal striatum, with dispersed positive cells in the cortex, while the same staining showed mostly scattered signal in the same brain regions in the B6 mice. Similar difference was observed in CNP immunofluorescence staining as well (**c**). **d**, **e** At P10 and P12, MBP signal continued to increase rapidly in both strains; however, it spread further into more superficial regions of the cortex and ventral striatum in the BTBR than the B6 brain. Similarly increased MBP signal in *female* BTBR brains during neonatal development was observed at P6 (**f**) and P8 (**g**). *n* = 2 to 4 for each strain at each age of each sex. White arrows in **a**, **f** indicate examples of MBP signal located in presumably myelin sheath that was starting to form. (**h**) CC1 (a specific marker for mature oligodendrocytes) and DAPI co-staining in external capsule region from P8 mice. White arrows indicate examples of CC1-positive cells. **i** Quantification of CC1-positive cells in the B6 and BTBR mice

Next, we probed myelin development using immunofluorescence staining of myelin basic protein (MBP), an integral component and specific marker of myelin^52–56^, whose cellular expression pattern has been widely used to characterize myelination process. We observed increased MBP signal in male BTBR brains at P6, compared with age- and sex-matched B6 mice. Close examination revealed that MBP signal was located in many cells in the BTBR frontal brain, including corpus callosum and dorsal striatum, and to a lesser extent, the cortex. Most of the positive cells displayed MBP signal in both cell bodies and numerous processes, with some already aligned in parallel tube-like structures presumably wrapping around axons. On the contrary, only a few MBP-positive cells could be identified in the same brain regions in the B6 brain, with the signal mostly restricted to the cell bodies (Fig. 2a). At P8, MBP signal in the BTBR frontal brain showed clear organization of myelin sheath in the corpus callosum and dorsal striatum, with dispersed positive cells in the cortex, while the same staining showed mostly scattered signal in the same brain regions in the B6 brain (Fig. 2b). A similar difference was observed in immunofluorescence staining of 2′,3′-cyclic nucleotide 3′-phosphodiesterase (CNP) as well (Fig. 2c). In older pups aged P10 (Fig. 2d) and P12 (Fig. 2e), MBP signal continued to increase rapidly in both mouse strains; however, it spread further into more dorsal regions of the cortex and ventral striatum in the BTBR than the B6 brain. Totally, we examined two to four mice of each strain at each age.

We also observed similar increased MBP signal in female BTBR brains during neonatal development. At P6, there were more cells with MBP-positive processes in the corpus callosum and dorsal striatum of the BTBR than the B6 brain (Fig. 2f). At P8, more widespread MBP signal was observed in the corpus callosum and dorsal striatum in the BTBR mice (Fig. 2g). Thus, both male and female BTBR mice had increased MBP-positive myelin structure during neonatal development.

Increased myelination suggested more abundant mature oligodendrocytes in the BTBR brain. Indeed, staining against CC1, a specific marker for mature oligodendrocytes, revealed that there was a significant increase in the number of positive cells in the BTBR mice compared with the B6 mice (Fig. 2h, i), corroborating the notion that myelination was elevated in the BTBR mice.

### Upregulated MBP expression accompanied increased myelination in neonatal BTBR mice

**Fig. 3:**
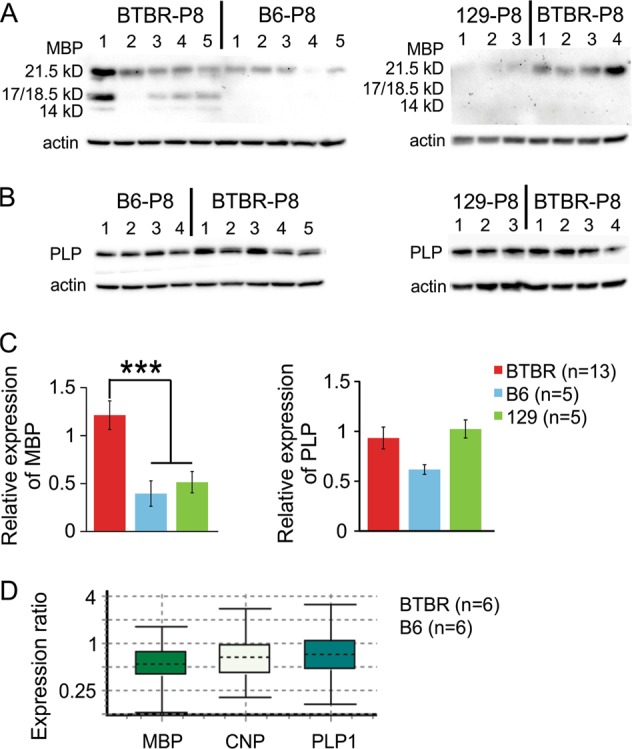
Upregulated MBP expression accompanied increased myelination. Western blot examining MBP (**a**) and PLP (**b**) protein expression in striatal homogenates from P8 mice. **c** Quantification of relative protein expression levels of MBP and PLP normalized by actin. **d** Relative mRNA expression levels of *MBP, CNP, and PLP1* in striatal homogenates from P8 mice, presented as a ratio of BTBR to B6 levels

As mentioned earlier, MBP is an essential component of mature compact myelin in the central nervous system, and has been considered as the ‘executive molecule of myelin’, serving multiple functions beyond structural support^52–56^. In addition, expression of MBP is a rate-limiting step in myelination in the central nervous system^52–56^. Here, we further quantified the protein expression level of MBP in the striatum, and found it was significantly increased in P8 male BTBR mice compared with two other age- and sex-matched inbred mouse strains: B6 and 129 (Fig. 3a, c). In contrast, the protein levels of myelin proteolipid protein (PLP), the most abundant protein of myelin in the central nervous system^57,58^, were similar among all three strains of mice (Fig. 3b, c), indicating that upregulated MBP protein expression is associated with the increased myelination observed in the BTBR mice. Further experiments indicated that there was no significant difference in the mRNA levels of myelin-related genes *MBP*, *CNP*, or *PLP1*, encoding MBP, CNP, or PLP, respectively, between the BTBR and B6 mice at P8 (Fig. 3d). These results suggest that the increased protein level of MBP in the BTBR mice is likely due to alterations in translation or post-translational processing, instead of transcription of the gene.

### Similar myelin pattern and thickness in adolescent and adult B6 and BTBR frontal brain

**Fig. 4:**
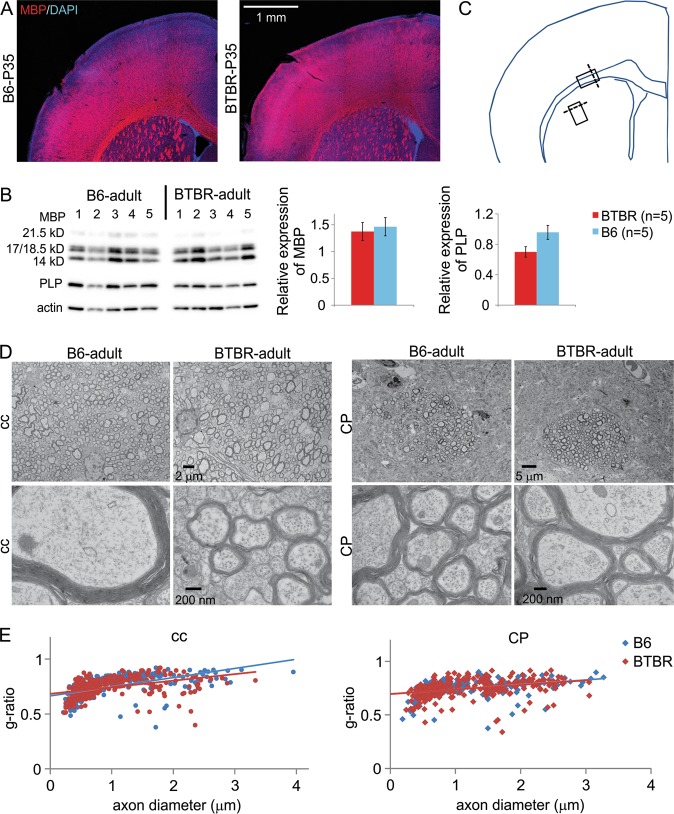
Similar myelin pattern and thickness in adolescent and adult B6 and BTBR frontal brain. **a** At P35, the pattern of MBP immunofluorescent signal was similar in the frontal brain between male B6 and BTBR mice. *n* = 4 mice for each strain. **b** Relative expression levels of striatal MBP and PLP proteins were also similar between B6 and BTBR adult male animals at 8–10 weeks old. **c** Schematic indicating the locations and regions where tissue was dissected (boxes), and the planes of tissue sectioning (dotted lines) for EM analysis. **d** Electron microscopy showed typical myelination pattern and myelin micro structure in corpus callosum (cc) and striatum (CP) from both adult male BTBR and B6 brains. Images of both lower (upper panels) and higher (lower panels) magnifications are shown. **e** The g-ratios of axons in both brain regions were similar between the B6 and BTBR mice. Totally, 316 axons in corpus callosum and 280 axons in striatum from four B6 mice, and 346 axons in corpus callosum and 291 axons in striatum from four BTBR mice were analyzed

We further followed myelin development using immunofluorescence staining of MBP into older ages. At P35, we did not observe any obvious difference in the frontal brain between male BTBR and B6 mice (Fig. 4a) (*n* = 4 mice for each strain). In addition, relative protein expression levels of MBP and PLP in the striatum were also similar between adult male BTBR and B6 animals (Fig. 4b). Using electron microscopy, we observed typical myelination pattern and myelin micro structure in corpus callosum and dorsal lateral striatum from both adult male BTBR and B6 brains (Fig. 4c, d). Furthermore, the g-ratios^50^ of axons in both brain regions were similar between the two strains, indicating that the relative thickness of myelin was not altered (Fig. 4e). Thus, increased myelination in the frontal brain of the BTBR mice only appeared during neonatal development; both BTBR and B6 mice reached similar degree of myelination at adult stage.

### PDGFRα signaling was reduced in the frontal brain of neonatal BTBR mice

**Fig. 5:**
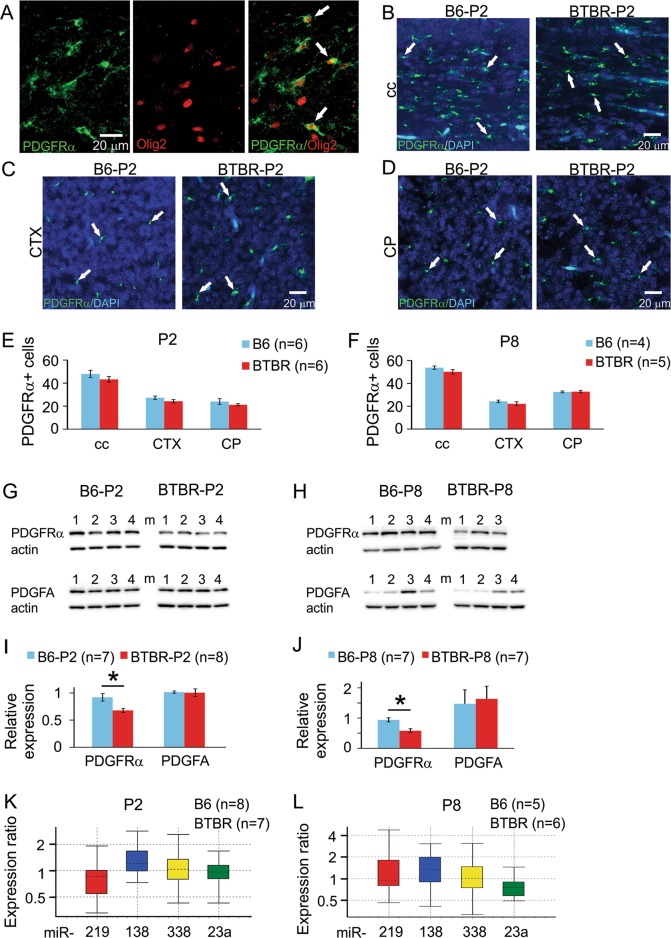
PDGFRα signaling was reduced in neonatal frontal brain of the BTBR mice. **a** Co-immunofluorescence staining of PDGFRα and Olig2 showed that PDGFRα-positive cells also expressed Olig2, another marker for oligodrendroglial lineage. White arrows indicate examples of cells that are positive for both PDGFRα and Olig2. **b**–**d** PDGFRα and DAPI co-staining for cell counting purpose in the corpus callosum (cc), somatosensory cortex (CTX), and striatum (CP). White arrows indicate examples of cells that are positive for PDGFRα. **e**, **f** Quantification of PDGFRα-positive cells at both P2 and P8 in the three brain regions. **g**, **h** Western blots showing the protein expression levels of PDGFRα and PDGFA at both P2 and P8. **i**, **j** Quantification of western blot results indicated that relative expression levels of PDGFRα were significantly reduced in the BTBR compared with the B6 mice at both P2 and P8, while relative expression levels of PDGFA were similar between the two strains of mice at both ages. **k**, **l** Relative levels of miR-219, 138, 338, and 23a in striatal homogenates from P2 and P8 mice, presented as a ratio of BTBR to B6 levels

Based on these data, we investigated PDGFRα signaling, as it is specifically expressed by oligodendrocyte precursor cells (OPCs)^59,60^ and critical in regulating the timing of their differentiation, such that it is down-regulated when OPCs stop proliferation to start differentiation. Extensive studies indicate that PDGFRα level is a negative regulator of the biological clock controlling the maturation process of OPCs^61–67^. Using immunofluorescence staining (Fig. 5a–d), we found that the number of PDGFRα-positive cells were slightly, but not significantly, decreased in the corpus callosum, somatosensory cortex, and dorsal striatum of the BTBR mice compared with the B6 mice at both P2 and P8 (Fig. 5e, f). Meanwhile, the protein expression level of PDGFRα (Fig. 5g, h) was significantly reduced in the frontal brain of the BTBR mice at both P2 and P8 (Fig. 5i, j), while the expression level of PDGFA, the ligand of PDGFRα involved in the timing of OPC differentiation, was similar between the two strains (Fig. 5g–j). Thus, PDGFRα signaling was reduced in the OPCs in the frontal brain of neonatal BTBR mice, consistent with accelerated myelin development observed in the same region.

Recent studies have uncovered important roles of microRNAs in regulating myelination during development^68–71^. Several microRNA species, including miR-219, 338, 138, and 23a, have been shown to inhibit expression of PDGFRα^72–74^. Thus, we tested whether changes in PDGFRα protein expression observed in the BTBR mice could be due to altered levels of these microRNAs. Quantitative RT-PCR of these microRNA species in the frontal brain showed that there was no difference in abundance between BTBR and B6 mice at both P2 and P8 (Fig. 5k, l), suggesting that these microRNAs are unlikely to contribute to the accelerated myelination observed in the BTBR mice.

## Discussion

### Development of myelin in ASD patients

Although neuroimaging studies on older children, adolescents, and adults with ASD have generally shown hypo-connectivity of brain circuits, more recent studies on young children (1–4 year old) have suggested the opposite may be the case, that is, hyper-connectivity during early neurodevelopment^6–15^. However, the cellular and molecular substrates of the hyper-connectivity remain poorly understood. In these studies, increased FA and in some cases greater volume were observed within several fiber tracts that innervate the frontal lobes, commissural fibers, long-range association tracts, and thalamocortical projection fibers in the ASD population, followed by overall slower than typical apparent rate of continued development. Notably, the relationship between diffusion and volume measures and severity of social and communication symptoms was dependent on the age at which the neuroimaging measurement took place: greater FA and volume of frontal tracts in early years predicted greater symptom severity later at the age of final diagnosis, whereas a trend toward the opposite relationship (lower FA and volume correlated with greater severity) was observed for frontal tracts measured at older ages, suggesting that greater abnormality in early growth may be associated with greater severity of symptoms observed later^6^. One possible reason of increased FA is greater extent of myelination. Thus, it could be reasoned that accelerated myelination could potentially contribute to the hyper-connectivity during early development of ASD, among other factors such as increased numbers of axons^23,75^.

Increased brain volume, especially in young children with ASD, has been a consistent finding^19,20,76^, resulting from both gray and white matter increases^6,15^. Although involvement of myelination in increased brain volume is not clear, it is possible that it also plays an important but so far underappreciated role.

Neuroimaging studies on myelin per se in ASD patients are very limited, due to technical limitations. Notably, a recent study utilized magnetization transfer imaging, which has been shown to indirectly represent myelination during development^77^, to compare 101 young children with autism (mean age = 4.5) and 35 typically developing children (mean age = 4.0), including both boys and girls. The magnetization transfer ratio (MTR) was calculated for each voxel within the midsagittal area of the corpus callosum. Mean MTR as well as MTR histogram peak height and location were significantly higher in children with autism, suggesting accelerated myelination of the corpus callosum in very young children with autism^30^.

Histological studies of postmortem patient brain tissue have been very rare, especially from samples of young children. Ultrastructural investigation of postmortem adult brain tissue revealed decreased myelin thickness in fibers beneath the orbitofrontal cortex^75^. It would be important to determine whether such abnormalities are present in young ASD population and how they might change over time during development.

The consequences of accelerated myelination during brain development are less well understood. Notably, accelerated myelination is frequently observed in infants with hemimegalencephaly, a rare neurological condition characterized by frequent seizures, usually followed by mental retardation^78^, both of which are comorbidities of ASD.

### Development of myelin in animal models of ASD

Studies on myelination using animal models of ASD have also been very limited. Thus far, these studies have found delayed myelination in general. One study reported a delay in myelination accompanied by reduced OPC number in the cerebellum, and smaller cerebellar volume in *Fmr1* knockout mice during early postnatal development. Interestingly, although the amounts of myelin specific proteins are reduced in neonatal mutant mice, they reach similar levels, or even surpass those observed in the wildtype control animals in adulthood^79^. Another study showed that maternal immune activation in mice delays myelination and axonal development in the hippocampus, but not cortex, of the offspring, and the deficits disappear when the animals reach the adult stage^80^. In addition, disruption of the tuberous sclerosis complex (TSC) leads to hypomyelination^81,82^. Interestingly, in a rat model of diffuse white matter injury, which is a major complication in preterm infants and associated with psychological conditions including ASD, delayed cortical myelination and ASD-like behaviors were observed^83^. Together, these studies suggest that different etiologies underlying ASD may affect development of myelin differently.

Here, our study showed precocious myelination in the frontal brain of the BTBR mouse model of ASD. According to previous studies, oligodendrocyte maturation and myelination in neonatal mice around P6 to P12 corresponds to term infants (36–40 week) in humans^84,85^, when the brain experiences peak growth spurt and gliogenesis, and the predominance of oligodendrocyte maturation state changes from pre-oligodendrocytes to immature oligodendrocytes. Our findings suggest accelerated myelin development could potentially contribute to hyper-connectivity and increased brain volume typically observed in young children with ASD. However, myelin pattern and thickness were unaltered in adult BTBR animals, suggesting a blunted developmental trajectory, reminiscent of what has been observed in ASD patients. Thus, precocious myelination in the BTBR mice could potentially serve as a model to investigate the mechanisms and consequences of atypical myelin development in ASD.

### Mechanisms underlying precocious myelination in the BTBR mice

MBP has long been recognized as a crucial component of mature myelin in the central nervous system. It is responsible for adhesion of the cytosolic surface between multiple layers of compact myelin. Mice harboring shiverer spontaneous mutation in the *MBP* gene have poorly formed myelin^53^. Further studies showed that the expression of MBP is a rate-limiting step in myelination in the central nervous system^54,55^. In contrast, ablation of other major myelin proteins such as PLP^57,58^ and CNP^86^ seems to induce secondary axonal consequences rather than affecting myelination process per se. Besides structural functions in myelin, MBP also actively participate in other processes during myelination, and thus has been referred to as the ‘executive molecule of myelin’^52,56^. The importance of MBP for myelination is supported by the notion that the emergence of MBP in ancient jawed fish conferred myelination ability, which sped up the velocity of nerve conduction more than 20-fold. Without MBP and myelin, jawed vertebrates as we know them, including humans, could not have evolved^87,88^. Interestingly, we observed an increase in MBP protein, but not the mRNA, expression levels in the BTBR mice (Fig. 3). As MBP is transported in oligodendrocytes as mRNA rather than protein, it has been suggested that localized translation of MBP is advantageous for rapid and axon-tailored synthesis of the molecule as the required amounts may vary at distinct axonal segments, and that localized translation of MBP is tightly regulated^89^. Thus, it is plausible that altered translation contributes to increased MBP protein levels in the BTBR mice. It was also observed that the protein levels of MBP appeared quite variable in P8 animals even among the same strain of mice (Fig. 3). A possible reason was that during neonatal development when MBP was just beginning to be expressed and increased rapidly, individual variations might be more than those when myelination had reached a stable level, as observed in adult animals (Fig. 4).

The role of PDGFRα in determining the timing of OPC differentiation has been extensively studied. PDGFRα is the only PDGF receptor isoform expressed on OPCs^59,60^. During OPC differentiation, expression of PDGFRα in OPCs progressively decreases and then is rapidly extinguished in mature oligodendrocytes^63,64^. PDGF is a major mitogen for OPC proliferation^65^, although PDGF signaling is unlikely the only mechanism determining the final numbers of oligodendrocyte^38^, as alterations of its levels do not affect the levels of myelin at maturity^90^. Meanwhile, PDGF signaling has also been shown to inhibit differentiation of OPCs into oligodendrocytes. Withdrawal of PDGF from culture medium triggers differentiation of OPCs^61,62,65^. Consistently, genetic ablation of PDGFRα also leads to precocious differentiation of OPCs both in vitro and in vivo, and accelerated myelination^66,67^. These results indicate that PDGFRα level is a negative regulator of the biological clock controlling the maturation process of OPCs. Thus, reduced PDGFRα signaling in neonatal BTBR brain could potentially result in the accelerated myelination we have observed.

Notably, studies using the BTBR mice have uncovered profound alterations in both structural and functional connectivity^32,36,91–96^, the most striking one being the non-crossing corpus callosum in these animals^31,36–38^. In addition, our previous work revealed impaired synaptic pruning and dendritic overgrowth during neonatal development in the BTBR brain^97,98^. The relation between these changes and the accelerated myelination observed here needs to be examined in the future. In addition, the total number of axons in white matter tracts in the BTBR mice has not been determined. Future studies to examine parameters such as whether, where, and to what degree axon number may alter could reveal additional axonal abnormalities in this mouse model.

In summary, this study revealed precocious myelination in a mouse model of autism. The high prevalence of ASD and the grave cost associated with psychological, physiological, and socioeconomic burden call for improved understanding of the early developmental pathophysiology, which may best reveal fundamental biological underpinnings of ASD, and would also assist early diagnosis and inform therapeutic options, as well as help to identify high-risk individuals.
